# Radiotherapy with Helium Ions Has the Potential to Improve Both Endocrine and Neurocognitive Outcome in Pediatric Patients with Ependymoma

**DOI:** 10.3390/cancers14235865

**Published:** 2022-11-28

**Authors:** Ricarda Wickert, Thomas Tessonnier, Maximilian Deng, Sebastian Adeberg, Katharina Seidensaal, Line Hoeltgen, Jürgen Debus, Klaus Herfarth, Semi B. Harrabi

**Affiliations:** 1 Department of Radiation Oncology, Heidelberg University Hospital, 69120 Heidelberg, Germany; 2 Heidelberg Institute of Radiation Oncology (HIRO), 69120 Heidelberg, Germany; 3 National Center for Tumor Diseases (NCT), 69120 Heidelberg, Germany; 4 Heidelberg Ion-Beam Therapy Center (HIT), Department of Radiation Oncology, Heidelberg University Hospital, 69120 Heidelberg, Germany; 5 Clinical Cooperation Unit Radiation Oncology, German Cancer Research Center (DKFZ), 69120 Heidelberg, Germany; 6 German Cancer Consortium (DKTK), 69120 Heidelberg, Germany

**Keywords:** pediatric brain tumors, ependymoma, IMRT, ion beam therapy, protons, helium ions, normal tissue complication probability, late sequelae

## Abstract

**Simple Summary:**

Radiotherapy is a mainstay in the treatment of pediatric ependymoma. It requires precise dose application and optimal protection of organs at risk to minimize long-term sequelae. Compared to photon radiation treatment, proton therapy often achieves even better results regarding target coverage and organ-sparing. Due to their physical properties, helium ions could even further reduce side effects, providing better protection of healthy tissue despite similar target coverage. The present study reports the first in-depth dosimetric assessment of scanned helium ion plans compared to both state-of the art scanned protons and intensity modulated radiotherapy. Furthermore, the rationale and potential benefit of helium ions is substantiated by normal tissue complication probability analyses.

**Abstract:**

Ependymomas are the third most-frequent pediatric brain tumors. To prevent local recurrence, the resection site should be irradiated. Compared to photon radiation treatment, proton therapy often achieves even better results regarding target coverage and organ-sparing. Due to their physical properties, helium ions could further reduce side effects, providing better protection of healthy tissue despite similar target coverage. In our in silico study, 15 pediatric ependymoma patients were considered. All patients underwent adjuvant radiotherapeutic treatment with active-scanned protons at Heidelberg Ion Beam Therapy Center (HIT). Both helium ion and highly conformal IMRT plans were calculated to evaluate the potential dosimetric advantage of ion beam therapy compared to the current state-of-the-art photon-based treatments. To estimate the potential clinical benefit of helium ions, normal tissue complication probabilities (NTCP) were calculated. Target coverage was comparable in all three modalities. As expected, the integral dose absorbed by healthy brain tissue could be significantly reduced with protons by up to −48% vs. IMRT. Even compared to actively scanned protons, relative dose reductions for critical neuronal structures of up to another −39% were achieved when using helium ions. The dose distribution of helium ions is significantly superior when compared to proton therapy and IMRT due to the improved sparing of OAR. In fact, previous studies could clearly demonstrate that the dosimetric advantage of protons translates into a measurable clinical benefit for pediatric patients with brain tumors. Given the dose–response relationship of critical organs at risk combined with NTCP calculation, the results of our study provide a strong rationale that the use of helium ions has the potential to even further reduce the risk for treatment related sequelae.

## 1. Introduction

**Figure 1:**
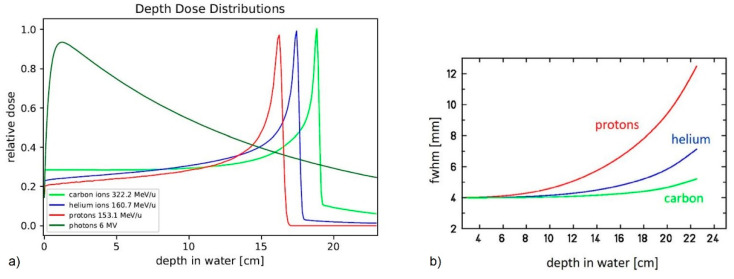
Physical properties of helium ions compared to photons and other ions. (**a**) relative absorbed dose of photons, protons, helium and carbon ions as a function of penetration depth. Bragg peak of helium ions is a sharper compared to protons. Fragmentation tail is smaller than for carbon ions. (**b**) full width half maximum of beams of protons, helium and carbon ions as a function of depth in water. Helium ions show less lateral scattering compared to protons (image: courtesy of U. Weber, GSI).

Among pediatric patients, ependymomas are the third most-common tumors of the central nervous system [1]. They originate from degenerated ependymal cells that, when healthy, line the ventricular system and the spinal canal. Most children receive their diagnosis in the first decade of life [2,3]. The broadly accepted primary treatment of ependymoma consists of tumor surgery. Hereby, achieving a complete resection is crucial for a good prognosis [4]. To achieve further disease control, radiotherapy (RT) plays an important role in the adjuvant treatment of ependymoma. So far, in absence of metastases, patients are given focal radiotherapy with doses from 54 to 59.4 Gy depending on age, number of performed surgeries and neurological status [5,6]. Due to improved treatment techniques regarding surgery, as well as radiotherapy, more and more children are expected to defeat their disease and become long-term cancer survivors. Unfortunately, longer life spans combined with the high radiation sensitivity of children are associated with higher risks for radiation-related long-term sequelae [7]. These include neurocognitive impairment, endocrine dysfunction, sensorineural hearing loss as well as vasculopathy and development of secondary malignancies [8,9,10,11]. To minimize the appearance probability of long-term consequences, healthy brain tissue and organs at risk (OAR) must be spared as much as possible. The implementation of active-scanned proton therapy (PRT) into clinical practice has changed the capabilities of radiotherapeutic treatment in terms of the precision of dose deposition and sparing of critical structures. Studies have already shown that the dosimetric advantages of protons in comparison to conformal radiotherapy with photons result in measurable clinical benefits for the patients [12,13]. The biophysical properties of charged particles allow a very precise and high conformal dose application (see Figure 1a). Theoretical considerations indicate that dose to healthy tissue can be further reduced by using slightly heavier ions—helium ions [14,15]. In contrast to protons, helium ions exhibit less lateral scattering (see Figure 1b). This leads to even steeper dose gradients. Additionally, helium ions and protons show the same ratio between mass and the squared nuclear charge number. In contrast to other heavy ions, such as carbon, helium ions thus yield the same range at the same energy per nucleon as protons [14]. Furthermore, the relative biological effectiveness (RBE) of helium ions is expected to be higher than the RBE of protons [16].

As neurocognitive impairment and endocrine dysfunction are the most common late-term effects in children after brain irradiation [17,18], the OAR important for these functions were considered in particular. These include the hippocampi as well as the pituitary gland and the inner ears. Quantifying the dosimetric advantages of helium ions also provides an opportunity to evaluate their potential clinical benefit with respect to long-term side effects. Hereby, so-called normal tissue complication probability (NTCP) models come into use. In contrast to clinical trials with long follow-up times, these models allow a much faster assessment of the risk of long-term sequelae [19,20]. The most frequently used models are the Lyman–Kutcher–Burman (LKB) model and the Relative-Seriality model. By inserting parameters which represent the potential biological damage caused by irradiation the patient specific risk for long-term side effects can be calculated [21]. In fact, NTCPs are expected to become gold standard for identifying the patients likely to benefit most from particle therapy [22]. Due to its limited availability and its high treatment costs, it is very important to define strategies for clinical decision making. In view of the possible dosimetric superiority of helium ion beam therapy compared to radiotherapy with protons or photons, this in-silico study was conducted to confirm and quantify the dose reductions for OAR. To further evaluate the dosimetric data from this study with regard to the clinical benefit of helium ions, the authors set out to compare NTCPs based on helium ion beam treatment plan data with NTCPs of PRT and conventional RT.

## 2. Materials and Methods

A total of 15 patients with histologically confirmed ependymoma treated at Heidelberg Ion Beam therapy Center (HIT) between 2019 and 2021 were considered for this in-silico study. All patients underwent an adjuvant radiotherapeutic treatment with actively scanned protons. Additional inclusion criteria were tumor localization in the posterior fossa. The median age was 4.7 years (range 1 to 21 years).

For each patient, both helium ion and highly conformal IMRT plans were calculated in addition to the available proton-based plans using the treatment-planning software RayStation (version 10A, Raysearch Laboratories AB, Stockholm, Sweden). The prescription dose was set at 54 Gy in 30 fractions of 1.8 Gy and was prescribed in the median dose of the CTV (D50%). Proton plans with a prescription dose of 59.4 Gy were adjusted to 54 Gy. Photon RT and helium ion RT planning was performed on the original planning CT data sets. Contouring of the OAR was performed from the clinical approved proton plans. In four cases the contouring of some of the considered OAR was missing and conducted retrospectively. The initial dose constraints of the proton plans were adapted and then used for calculating the helium-based plans. Photon plans were designed according to guidelines of the authors’ institution. Helium ion and photon plans both were optimized for each patient. Thereby, 95% of the target volume received at least 95% of the prescription dose. All treatment plans were reviewed by radio-oncologists experienced in the field of pediatric radiotherapy.

For biological dose optimization, the modified microdosimetric kinetic model (mMKM) was used with the clinical parameters as described before [23]. All the dose specifications of helium ions are considered as biological doses (GyRBE).

Qualitative and quantitative dose evaluations were performed for all three treatment modalities. Dose volume histograms were plotted for each region of interest (ROI). Additionally, dosimetric parameters were extracted to check for appropriate target volume coverage and assure compliance with the given dose constraints. CTV coverage was assessed, calculating the average dose D_mean_, as well as D_1_, D_95_ and D_99_, which represent the dose values that receive 1%, 95% and 99% of the CTV, respectively. By calculating the homogeneity indices (HI) and inhomogeneity coefficients (IC), CTV coverage was further evaluated.

(1) $$\mathrm{HI}=\frac{\;D5-\;D95}{\mathrm{Dp}}\times 100$$

(2) $$\mathrm{IC}=\frac{\mathrm{Dmax}\;-\;\mathrm{Dmin}}{\mathrm{Dmean}}$$

D_5_ corresponds to the minimum dose in 5% of the CTV. D_p_ equates the dose prescribed to the CTV. The closer the HI is to zero, the more homogeneous is the target volume coverage [24]. The IC estimates the distribution variance of the CTV. Higher values indicate greater variability [25]. D_max_ and D_min_ represent the dose values that are received from the largest and smallest percentages of the CTV, respectively. With respect to the OAR, the dosimetric parameters D_mean_, D_1_ and D_50_ were calculated to analyze the dose distribution. Thereby, D_50_ represents the dose received by 50% of the volume. In order to assess the total energy absorbed by a tissue volume during irradiation the integral dose (ID) was computed:

(3) $$\mathrm{ID}=\sum_{i}^{\;}D_{\mathrm{mean}}\times {\;V}_{i}$$

The ID is defined as the sum of the mean dose D_mean_ multiplied by the volume assuming that the voxels are of equal size and the density of the organ is uniform. ID also represents the area under the DVH [26]. D_mean_ represents the average dose of the considered ROI whereas V_i_ stands for the volume in cm^3^. For this analysis the simplified formula D_mean_ × V was used.

Normal tissue complication probability (NTCP) models are used to either predict the risk of toxicity stemming from radiotherapy or in order to compare, qualitatively, two radiation modalities on the possible patient adverse events. The model selected for this work was the Lyman–Kutcher–Burman model (LKB), which is one of the main models used clinically for NTCP modeling [27]. The LKB model takes as input the TD_50_(v), corresponding to the tolerance dose received for a relative volume v; the TD_50_ corresponding to the tolerance dose at which the organ of interest reaches a 50% complication probability; m, linked to the dose–response slope of the NTCP function; n, the parameter taking into account the volume effect of the irradiation. D corresponds to the dose received uniformly by the volume v of the structure.

(4) $$\mathrm{NTCP}=\frac{1}{\surd 2\pi }\int_{-\infty }^{t}e^{\frac{-x^{2}}{2}}\;dx$$

(5) $$t=\frac{D\;-{\;\mathrm{TD}}_{50}\left(v\right)\;}{m\;\times {\;\mathrm{TD}}_{50}\left(v\right)}$$

(6) $${\mathrm{TD}}_{50}\left(v\right)={\mathrm{TD}}_{50}\times v^{-n}$$

Equations (5) and (6) are further simplified using the generalized equivalent uniform dose (gEUD) formalism [28]:

(7) gEUD=[∑i(viDi1n)]n

With v_i_, corresponding to a volume fraction of a structure receiving a dose D_i_. Then Equation (5) can be rewritten as:

(8) $$t=\frac{\mathrm{gEUD}-{\mathrm{TD}}_{50}\;}{m\;\times {\;\mathrm{TD}}_{50}}$$

In order to compute the LKB for the different radiation modalities, the individual parameter TD_50_, m, n need to be known for the organs/structures of interests and the corresponding endpoint [21,28]. The LKB parameters used in this work are presented in Table 1. The dose volume histograms (DVH) of the different structures were extracted from the treatment planning system RayStation for NTCP computation in MATLAB (The MathWorks Inc., Natick, MA, USA).

All DVH data were exported as anonymized dvh-files. Then, the files were imported in in the web-based, interactive computing notebook environment Jupyter Notebook. Data analysis was performed using the programming language Python. Dosimetric parameters that were less than 1 Gy for all treatment modalities were not considered for the calculation of relative differences.

The study was approved by the Ethics Committee of the University Hospital Heidelberg.

Statistical analysis was carried out in the web-based, interactive computing notebook environment Jupyter Notebook using the programming language Python. The Friedman test with the Wilcoxon signed-ranked post-test was applied with corresponding two-sided 95% confidence intervals. A *p*-value < 0.05 was considered statistically significant. Dosimetric parameters that were less than 1 Gy for all treatment modalities were not considered in the statistical analysis.

## 3. Results

### 3.1. CTV Coverage

**Figure 2:**
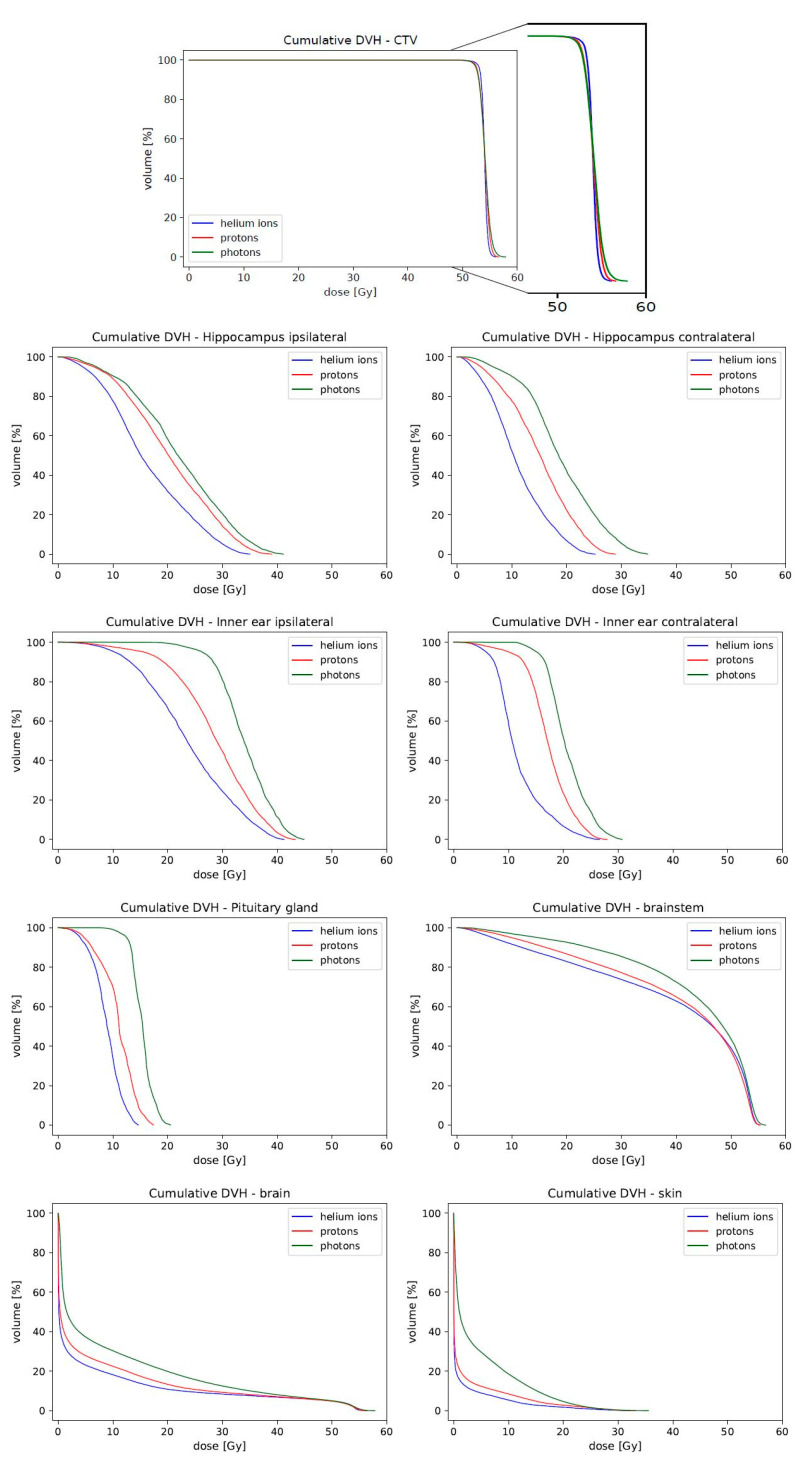
Cumulative dose–volume histograms (n = 15). Shown are the average sum doses of CTV and all OAR for helium ions (blue), protons (red) and photons (green). Target coverage is comparable. The dose–volume curve of helium ions is slightly steeper compared to the other treatment modalities. The dosimetric advantage of helium ions can be seen for all OAR.

Target volume coverage was comparable in all three treatment modalities. There was no significant difference in D_mean_ or IC. Comparing helium ions to protons, the D_1_ and the D_95_ differed significantly (Table 2). The steeper dose–volume curve of helium ions can also be identified in the cumulative dose–volume histogram (Figure 2). In addition, the HI of helium ions showed a significant deviation, indicating a higher homogeneity.

### 3.2. Sparing Organs at Risk

**Figure 3:**
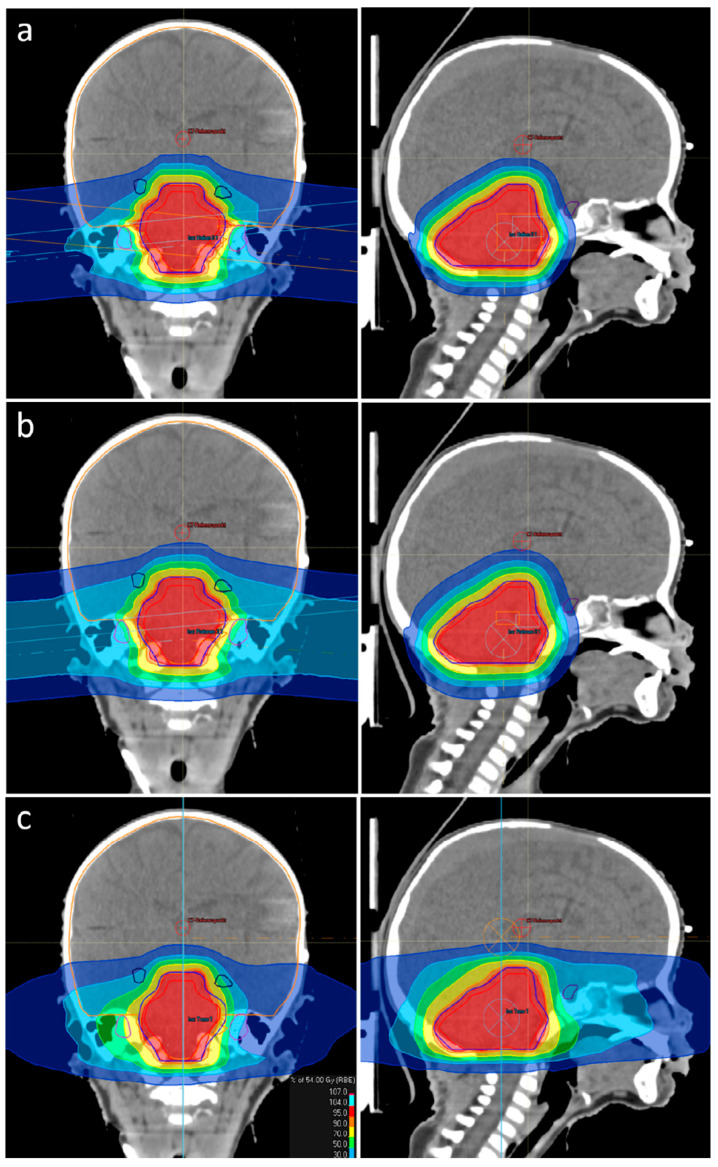
Comparison of dose distribution for a patient with ependymoma. (**a**) Helium ion beam therapy plan; (**b**) Proton beam therapy plan; (**c**) Helical intensity modulated radiotherapy plan. CTV is delineated in red, the pituitary gland in violet, the inner ears in purple and the hippocampi in dark blue. The treatment plan based on helium ions shows the best sparing of OAR due to steeper dose gradients.

OAR that play an important role in terms of neurocognitive and endocrine functions were assessed to compare all three treatment modalities. Figure 2 shows the cumulative DVH of the considered OAR. All OAR doses were lowest for helium ions followed by protons. How the steeper dose gradients of helium ions manifest themselves with respect to the irradiation fields can be seen in Figure 3.

The delineated OAR are best spared in the treatment plan based on helium ions. To further quantify the dose differences, dosimetric parameters were calculated for each OAR (see Table 2). Given the dosimetric advantage of protons over photons already demonstrated in previous studies, we directly compared only the dosimetric parameters of helium ions and protons. The comparison of protons and photons serves to validate the method used. Helium ion plans allowed for the significant sparing of the ipsilateral (IL) and contralateral (CL) hippocampus. Here, the D_1_, D_50_ and ID were decreased by −17% ± 6%, −33% ± 6% and −27% ± 6% IL, −22% ± 5%, −40% ± 6% and −36% ± 5% CL, respectively. The comparison of protons and photons showed no significant dose differences for the IL hippocampus and the D_50_ CL. In contrast, the D_mean_ and the D_1_ of the CL hippocampus were reduced by −29% ± 8% and −27% ± 7%, respectively.

Similar results were seen for critical IL and CL auditory organs. Although the difference of D_1_ when comparing helium ions to protons was not statistically significant, the D_50_ and ID were reduced by −26% ± 6% and −24% ± 6% IL, −42% ± 6% and −38% ± 6% CL, respectively. The D_1_ CL in helium ion plans was −18% ± 6% lower than in proton plans. For protons vs. photons there were no significant dose reductions for the CL inner ear. Statistically relevant differences were seen for D_50_ and ID IL. PRT allowed for dose reductions of −17% ± 10% and −17% ± 9%.

Findings for the centrally located pituitary gland reached statistical significance for both comparisons. Comparing helium ions to protons, the D_mean_, D_1_ and D_50_ were reduced by −39% ± 7%, −33% ± 9% and −40% ± 7%, respectively. The dose differences between the proton and photons plans were higher. PRT reduced the D_mean_, D_1_ and D_50_ by −48% ± 16%, −41% ± 13% and −48% ± 17%, respectively.

Regarding the brainstem, the reached dose reductions were lower than for the above-mentioned OAR in both comparisons. ID was reduced by −5% ± 2% (He vs. H^+^) and −9% ± 4% (H^+^ vs. Ph), respectively. No significance was found for differences of D_1_ and D_50_ in helium vs. proton plans. Differences in D_50_ were not significant for protons vs. photons as well.

Being an indicator for the conformity of the irradiation field, the ID to total brain tissue was significantly reduced by −15% ± 1% (He vs. H^+^) and −27% ± 2% (H^+^ vs. Ph), respectively. Analyzing the D_1_, values were −0.3% ± 0.1% lower for helium ions compared to protons and −0.4% ± 0.1% lower for protons vs. photons, respectively.

Regarding the skin, findings showed also statistical significance. D_1_ and ID differed by −14% ± 3% and −31% ± 2% (He vs. H^+^), respectively, and by −14% ± 4% and −58% ± 3% (H^+^ vs. Ph). For the D_50_ of the brain and skin, more than five of the patients’ values were close to zero for all treatment modalities. This made a relative comparison not applicable.

### 3.3. NTCP—Normal Tissue Complication Probabilty

NTCP calculation was performed for the endpoints endocrine dysfunction, tinnitus and hearing loss. For each endpoint the average NTCP was lowest for helium ions (Table 1).

**Figure 4:**
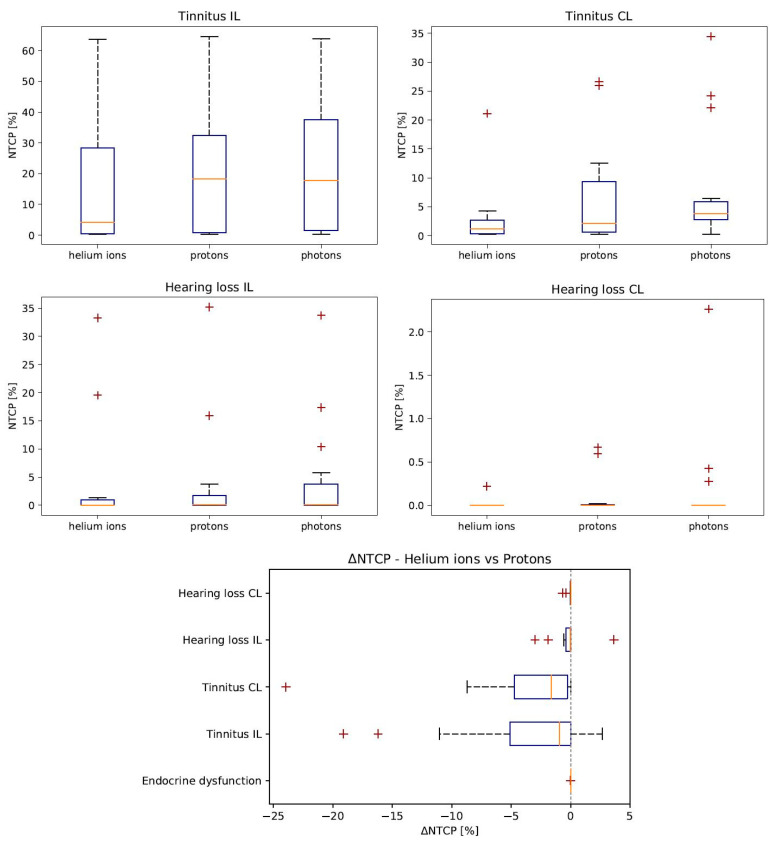
NTCP distribution for endpoints tinnitus and hearing loss (n = 15). The orange line shows the median. The blue box contains 50% of the calculated values. Helium ions lead to the lowest NTCP. Last boxplot shows ΔNTCP comparing helium ions to protons (NTCPHe-NTCPH+). Helium ions lead to NTCP reduction.

Regarding the pituitary gland, NTCP was close to zero for all three treatment modalities. With respect to the endpoint tinnitus, IL absolute differences of −4% ± 2% could be found when comparing helium ions to protons. A similar absolute difference of NTCP was seen for the CL side. The NTCP for hearing loss was lower than for tinnitus. NTCP values decreased IL from 5% ± 2% for photons to 4% ± 2% for protons and to 4% ± 2% for helium ions. In this case, the NTCP difference between helium ions and protons averaged −0.2% ± 0.4%. With regard to hearing loss, CL the calculated NTCP was less than 0.2% for all treatment modalities. Figure 4 also demonstrates the advantage of helium ions. The greatest impact of dose reduction could be found for the endpoint tinnitus.

## 4. Discussion

This study helps to quantify the dosimetric properties of helium ion beam therapy. The data present a distinct dosimetric advantage of helium ions compared to PRT and conventional RT in terms of sparing organs at risk. Not only did the average dose, Dmean, show a significant dose decrease for the considered OAR with dose reductions up to −39%, but also the maximal dose D_1_ as well as the median dose D_50_ and the ID were significantly reduced by using helium ions. Dose reductions were particularly high for OAR with important functions regarding neurocognitive and endocrine outcomes. Here, dose differences were in a range of −14% to −42% including all considered dosimetric parameters. With respect to paired organs, especially the contralateral side was more spared, receiving a dose reduction 10–15 % higher than the ipsilateral side. Furthermore, NTCP calculations indicated a superior clinical benefit for patients when using helium ions. However, to what extent the observed dose reductions manifest clinically remains proven by long-term observation studies. Actually, the re-introduction of helium ion therapy into clinical routine is still in its infancy. In 2021, the first patient was irradiated using actively scanned helium ions at Heidelberg Ion Beam Therapy Center (HIT). It will be some time before this form of therapy is approved for widespread use and also for children. Until then, it is very important to identify patient collectives or tumor entities that will benefit the most from the limited particle therapy available.

In fact, the reports that include treatment planning comparisons for helium ions are very rare. In 2016, Knäusel et al. [29] analyzed the dosimetric advantages of helium ions compared to protons for pediatric patients with various tumor entities. They found that the dosimetric benefit of helium ions depended on indication and tumor geometries. For patients with neuroblastoma or ependymoma, significant dose reductions were seen. With respect to ependymoma, the median dose D_50_ of OAR was reduced up to 1.7 Gy concerning the pituitary gland.

The results were lower than expected from literature. In contrast, the data of our study are more in line with expectations and show higher dose reductions when using helium ions. One of the reasons for this deviation is certainly that in recent years the biophysical models on which treatment plan calculations are based have been further improved [15,30,31,32]. In their treatment planning comparison, including five meningioma patients, Tessonnier et al. [15] found dose reductions up to 7.8 Gy for D_50_ of the optic system comparing helium ions to protons. Overall, the results presented in this study confirmed superior OAR-sparing using helium ions for radiotherapeutic treatment.

With respect to the target coverage, both studies mentioned above report similar target volume coverage when comparing helium ions and protons. One of the meningioma cases even showed a better coverage. A larger D_95_ and a smaller D_5_ resulted in a sharper target volume DVH. Our data present comparable results, indicating a higher dose homogeneity. In fact, more precise and homogeneous target coverage allows for a further dose escalation in the tumorous tissue. Higher doses to the tumor are considered more effective in terms of disease control [5]. In this regard, Ager et al. reported that increased irradiation doses were associated with improved overall survival in ependymoma patients aged 2–18 years [33].

As previously mentioned, it is not yet proven by clinical trials whether the dosimetric advantages of helium ions directly translate into clinical benefits for treated patients. Given the dose–response relationships of the OAR, the results of our study suggest that helium ions clearly have the potential to further reduce the risk of irradiation related sequelae. So far, no clinically validated data exist to adjust existing NTCP models specifically to the effect of helium ions. However, studies that compare the clinical outcome of patients after proton or photon irradiation support the rationale that similar biological effects are caused when using the same fractionation and dose per fraction as compared to a photon dose. Kahalley et al. [12] examined the intellectual outcome of 79 patients with medulloblastoma. The average length of follow-up was 4.3 years. Proton therapy was associated with a superior neurocognitive outcome in most of the tested domains. No significant differences were seen for verbal reasoning and processing speed. In addition, Child et al. [34] found that focal radiotherapy using protons led to favorable long-term cognitive and academic outcomes in pediatric patients. Focally irradiated children generally showed similar cognitive status to a normalized sample of children with normal development. However, mild impairments concerning processing speed, fine motoric and academic fluency skills were detected. Nonetheless, these results provide a strong argument that patients, such as ependymoma patients, who require focal radiotherapy will extremely benefit from particle therapy, particularly helium ion-based therapy. Furthermore, a study published by Vatner et al. [13] modeled the effect of the radiation dose received by the pituitary and hypothalamus on endocrine outcomes in pediatric and young adult patients with brain tumors. Among other predictors of endocrinopathy, dose reduction plays one of the main roles with respect to endocrine sequelae. For example, if the total dose to pituitary and hypothalamus was reduced from ≥40 Gy to ≤20 Gy the actuarial incidence rate for growth hormone deficiency at 5 years decreased from 79% to 9%. The results suggest that a further dose reduction provided by helium ion beam therapy could lead to even better clinical outcomes.

However, looking at study results demonstrating the clinical benefit of protons only allows a qualitative classification of the clinical benefit of helium ions. That is where NTCP models come into play. In fact, the NTCP results of our study provide a first quantification of the risk of long-term sequelae. Although our NTCP calculations showed results in favor of particle and especially helium ions beam therapy, the values are to be taken with caution. To obtain the endpoint-specific parameters for the calculation, the NTCP models are trained using data with known clinical outcomes after radiotherapy treatment. These data sets are mostly based on largely outdated data of adult patients who received conventional radiotherapy [21]. With currently available model parameters, NTCP calculations may not be appropriate for pediatric patients or for helium ions. In addition, NTCP modulation is very complex. It depends on various factors such as age and sex [21]. Further research is needed to develop parameters that are more appropriate for specific patient populations. Lastly, since NTCP values are only predictive for the suffering of side-effects, long-term clinical trials are still needed to confirm the superior outcome of helium ion-based RT.

Although the present findings suggest strong evidence in favor of helium ion beam therapy, the limitations of our study should be considered. First of all, sparing of OAR was only analyzed for a few organs at risk. A detailed analysis of further OAR such as the chiasma, optic system and ventricular system would also be interesting. With respect to the neurocognitive and endocrine outcomes, it would be very important to additionally assess differences in dose distributions of the thalamus, the amygdala, the subventricular zone and the hypothalamus [35,36]. Second, for NTCP calculation we used model parameters that were based on adult patients’ data except for the pituitary gland. Also, only endpoints for endocrine and auditory function were calculated. Uniform NTCP parameters for endpoints related to the hippocampus could not be found in the literature. Nevertheless, we see our results as first approach preserving a general trend in favor of the clinical benefit of helium ions in the treatment of pediatric patients.

Furthermore, a case number of 15 patients is relatively low. Following studies should be conducted with more patient data. However, this study affirms the expected dosimetric potential of helium ions and provides groundwork for future studies.

Another point to mention is that the prescription dose was limited to 54 Gy in order to achieve better comparability. In fact, depending on age, the number of performed surgeries and neurocognitive status, for pediatric ependymoma patients a dose of 59.4 Gy is generally recommended [5]. Such high doses require the additional sparing of the spinal cord and the brainstem as recommended for the currently ongoing SIOP Ependymoma II trial [6]. However, with a prescription dose of 59.4 Gy, the necessary dose constraints would have prevented the comparison of established indices. Actually, our results based on a target dose of 54 Gy underline the potential of helium ions even more as it can be assumed that the measured benefits are even greater at higher doses.

The strength of this study is being one of the first treatment plan comparisons for helium ions, protons and photons. Furthermore, the results confirm the dosimetric advantages of helium ions and thus provide a basis for further studies. Our findings suggest that the distinct dosimetric potential of helium ions in terms of dose reduction definitely comes into effect when treating pediatric patients with ependymoma. Hence, our results help to identify patient collectives who are particularly suitable for particle therapy, especially helium ion-based therapy. In addition, the first calculations of NTCP based on helium ion treatment plans were performed within this study. That not only allows further quantification of the clinical benefit of helium ions compared to protons and photons, but also brings the use of NTCP models closer to clinical routine.

## 5. Conclusions

Dose distribution of helium ions is significantly superior when compared to proton therapy and conventional radiotherapy. OAR that are considered essential for neurocognitive and endocrine function and thereby play an important role regarding the quality of life are better spared. The target coverage of all treatment modalities are comparable. In fact, helium ions show a slightly more precise and homogenous target coverage. Previous studies confirmed that dosimetric advantages of proton therapy translated into better clinical outcomes in comparison with conventional radiotherapy. Given the dose–response relationship of OAR combined with the results of NTCP calculation, our study gives a strong indication that helium ions lead to further reduction of treatment-related side effects. This makes them a superior alternative to proton and photon irradiation, especially for the treatment of pediatric patients with ependymoma.

## Figures and Tables

**Table 1:** NTCP parameters and comparison of NTCP.

Structure	Endpoint	TD_50_ [Gy]	m	n	
Cochlea [21]	Tinnitus	46.52	0.35	1	
Cochlea [21]	Hearing loss	55.57	0.14	1	
Pituitary [27]	Endocrine dysfunction	60.6	0.08	1	
Endpoint	helium	protons	photons	He vs. H^+^	H^+^ vs. Ph
				absolute difference	
Endocrine dysfunction	0.0006 ± 0.0004	0.003 ± 0.002	0.006 ± 0.006	−0.002 ± 0.002	−0.003 ± 0.004
Tinnitus Chochlea IL	16 ± 5	20 ± 5	23 ± 6	−4 ± 2	−3 ± 1
Tinnitus Cochlea CL	3 ± 1	7 ± 2	8 ± 3	−4 ± 2	−1 ± 2
Hearing loss Chochlea IL	4 ± 2	4 ± 2	5 ± 2	−0.2 ± 0.4	−0.8 ± 0.5
Hearing loss Chochlea CL	0.01 ± 0.01	0.09 ± 0.06	0.2 ± 0.2	−0.07 ± 0.05	−0.1 ± 0.1

Note: The top of the table shows the parameters used for NTCP calculation. NTCP values are given as a percentage as mean values with error of the mean (n = 15).

**Table 2:** Dose comparison target volume and OAR.

CTV	Helium Ions		Protons	Photons	He vs. H^+^	H^+^ vs. Ph
					rel. difference [%]	rel. difference [%]
D_mean_	54.00 ± 0.03		54.03 ± 0.03	54.11 ± 0.03	−0.07 ± 0.06	−0.15 ± 0.06 *
D_1_	55.3 ± 0.1		55.8 ± 0.1	56.5 ± 0.2	−0.9 ± 0.3 *	−1.2 ± 0.4 *
D_95_	53.1 ± 0.1		52.7 ± 0.1	52.61 ± 0.09	0.8 ± 0.2 *	0.1 ± 0.3
D_99_	52.3 ± 0.2		51.9 ± 0.2	51.9 ± 0.1	0.6 ± 0.2	0.2 ± 0.4
HI	3.2 ± 0.3		4.8 ± 0.3	5.6 ± 0.3	−33 ± 7 *	−12 ± 8
IC	0.14 ± 0.01		0.14 ± 0.01	0.149 ± 0.008	1 ± 9	−3 ± 9
OAR		helium ions	protons	photons	He vs. H^+^	H^+^ vs. Ph
					Rel. difference [%]	Rel. difference [%]
Hippocampus	D_mean_	17 ± 3	21 ± 3	22 ± 3	−27 ± 6 *	−4 ± 9
ipsilateral	D_1_	32 ± 5	36 ± 4	38 ± 4	−17 ± 6 *	−1 ± 10
	D_50_	15 ± 3	20 ± 3	21 ± 3	−33 ± 6 *	8 ± 20
	ID	23 ± 3	28 ± 3	31 ± 4	−27 ± 6 *	−4 ± 9
Hippocampus	D_mean_	11 ± 3	15 ± 3	19 ± 3	−36 ± 5 *	−29 ± 8 *
contralateral	D_1_	22 ± 5	26 ± 4	33 ± 4	−22 ± 5 *	−27 ± 7 *
	D_50_	11 ± 2	15 ± 3	18 ± 3	−40 ± 6 *	−20 ± 10
	ID	15 ± 3	20 ± 4	27 ± 5	−36 ± 5 *	−29 ± 8 *
Pituitary	D_mean_	9 ± 4	12 ± 4	15 ± 3	−39 ± 7 *	−48 ± 16 *
	D_1_	14 ± 5	16 ± 5	19 ± 4	−33 ± 8 *	−41 ± 13 *
	D_50_	9 ± 4	12 ± 4	15 ± 3	−40 ± 7 *	−48 ± 17 *
	ID	1.5 ± 0.5	1.9 ± 0.6	2.9 ± 0.5	−39 ± 7 *	−48 ± 16 *
Inner ear	D_mean_	25 ± 4	29 ± 4	34 ± 3	−24 ± 6 *	−17 ± 9 *
ipsilateral	D_1_	38 ± 5	41 ± 4	43 ± 4	−14 ± 6	−6 ± 10
	D_50_	23 ± 4	29 ± 4	34 ± 3	−26 ± 6 *	−17 ± 10 *
	ID	27 ± 5	32 ± 5	36 ± 4	−24 ± 6 *	−17 ± 9 *
Inner ear	D_mean_	12 ± 3	18 ± 3	21 ± 3	−38 ± 6 *	−19 ± 12
contralateral	D_1_	23 ± 4	25 ± 5	29 ± 3	−18 ± 6 *	−18 ± 12
	D_50_	11 ± 2	17 ± 3	20 ± 2	−42 ± 6 *	−18 ± 12
	ID	12 ± 3	19 ± 3	21 ± 3	−38 ± 6 *	−19 ± 12
Brainstem	D_mean_	39 ± 3	41 ± 3	44 ± 2	−5 ± 2 *	−9 ± 4 *
	D_1_	54.5 ± 0.3	54.3 ± 0.3	55.1 ± 0.3	0.3 ± 0.4	−1.4 ± 0.6 *
	D_50_	44 ± 4	44 ± 3	46 ± 3	−5 ± 4	−9 ± 6
	ID	752 ± 55	776 ± 51	843 ± 48	−5 ± 2 *	−9 ± 4 *
Brain	D_mean_	6.4 ± 0.6	7.5 ± 0.7	10.1 ± 0.7	−15 ± 1 *	−27 ± 2 *
	D_1_	54.19 ± 0.07	54.4 ± 0.1	54.6 ± 0.1	−0.3 ± 0.1 *	−0.4 ± 0.1 *
	D_50_	0.14 ± 0.05	0.5 ± 0.3	1.9 ± 0.4	nan ± nan	−74 ± 10 *
	ID	8294 ± 614	9712 ± 716	13,325 ± 855	−15 ± 1 *	−27 ± 2 *

Note: D_mean_, D_1_, D_95_ and D_99_ and D_50_ in Gy, ID in Gy cm^3^. * indicates statistically significance with *p*-value < 0.05. Values are given as mean values with error of the mean (n = 15). When nan (not a number) appears in the table, dosimetric parameters were close to zero for all treatment modalities in more than five cases which made relative comparison not applicable.

## Data Availability

The data presented in this study are available on request from the corresponding author. The data are not publicly available.
